# Development of a Carprofen-Loaded Nanoemulsion for Topical Management of Post-Surgical Inflammation

**DOI:** 10.3390/pharmaceutics18060672

**Published:** 2026-05-29

**Authors:** Ayesha Bibi Feroze-Bakht, Lupe Carolina Espinoza, Lilian Sosa, Mireya Zelaya, Dagmar Gualotuña Campoverde, Jorge Morillo-Poma, Marcelle Silva-Abreu, Délia Chaves Moreira dos Santos, Antonio J. Braza, Ana Cristina Calpena

**Affiliations:** 1 Department of Pharmacy and Pharmaceutical Technology and Physical Chemistry, Faculty of Pharmacy and Food Sciences, University of Barcelona, 08028 Barcelona, Spain; ayeshaabdullah786@gmail.com (A.B.F.-B.); braza@ub.edu (A.J.B.); anacalpena@ub.edu (A.C.C.); 2 Department of Chemistry, Universidad Técnica Particular de Loja, Loja 110107, Ecuador; lcespinoza@utpl.edu.ec (L.C.E.); degualotuna1@utpl.edu.ec (D.G.C.); jrmorillo1@utpl.edu.ec (J.M.-P.); 3 Institute of Microbiological Research (IIM), Faculty of Sciences, National Autonomous University of Honduras (UNAH), Tegucigalpa 11101, Honduras; 4 Faculty of Chemical Sciences and Pharmacy, National Autonomous University of Honduras (UNAH), Tegucigalpa 11101, Honduras; 5 Laboratory of Histological Techniques, Faculty of Sciences, National Autonomous University of Honduras (UNAH), Tegucigalpa 11101, Honduras; mireya.zelaya@unah.edu.hn; 6 Institute of Nanoscience and Nanotechnology, University of Barcelona (UB), Av. Diagonal 645, 08028 Barcelona, Spain; 7 Department of Pharmacy and Nutrition, Federal University of Espírito Santo, Alto Universitario, S/N, Alegre 29500-000, ES, Brazil; delia.santos@ufes.br

**Keywords:** carprofen, nanoemulsion, drug delivery systems, inflammation

## Abstract

**Background/Objectives:** Carprofen (CP) is a potent non-steroidal anti-inflammatory drug whose clinical use is limited by systemic adverse effects associated with oral administration. The aim of this study was to develop and evaluate a CP-loaded nanoemulsion (CP-NE) as a topical formulation for the management of post-surgical inflammation in veterinary applications. **Methods:** CP-NE was physicochemically characterized in terms of droplet size, polydispersity index, morphology, pH, rheological behavior, spreadability, and stability. Biopharmaceutical performance was assessed through in vitro drug release and ex vivo permeation studies using porcine ear skin. Safety was evaluated using in vitro cytotoxicity assays in HaCaT keratinocytes, histological analysis of ex vivo porcine skin, and assessment of biomechanical skin parameters in mice. Finally, anti-inflammatory efficacy was investigated in a murine model. **Results:** CP-NE showed a mean droplet size of approximately 140 nm, low polydispersity, spherical morphology, and Newtonian flow behavior with good spreadability. Stability studies confirmed the absence of significant physical destabilization and acceptable chemical stability under refrigerated and room temperature conditions. Release studies demonstrated sustained drug release, while permeation assays revealed low systemic exposure and high drug retention within the skin. Safety evaluations indicated good biocompatibility with no cytotoxicity, no histological alterations in skin tissue, and no alteration of the skin’s biomechanical properties in volunteers. In vivo efficacy studies showed that CP-NE significantly reduced post-surgical inflammation, promoting faster restoration of skin architecture and improved wound appearance. **Conclusions:** These findings suggest that CP-NE represents a promising topical delivery system for localized anti-inflammatory therapy following surgical procedures, offering significant potential for veterinary applications.

## 1. Introduction

Inflammation is a fundamental defense mechanism characterized by dynamic alterations in blood flow, vascular permeability, and the coordinated recruitment of fluids, proteins, and leukocytes from the circulation system to damaged sites, ultimately designed to protect living tissues and enhance tissue repair [1,2]. Surgical injuries, evoked during surgical interventions, inherently induce this response through surgical trauma, anesthesia interventions, and stress. As a consequence, there is an endocrine alteration, an increase in plasma cortisol, and activation of the sympathetic nervous system, collectively initiating the systemic inflammatory cascade [3,4]. The postoperative inflammatory reaction involves a substantial increase in proinflammatory cytokines, including interleukin (IL)-1β, IL-6, and tumor necrosis factor α (TNF-α). These cytokines are integral to the coordinated cellular process of wound healing, promoting the migration and proliferation of neutrophils and macrophages at the wound site to eliminate pathogens, remove necrotic tissue, and initiate tissue regeneration [5]. Although post-surgical inflammation is a critical physiological response for effective healing, excessive or prolonged inflammation can adversely affect wound healing, leading to postoperative complications, such as chronic inflammation and impaired tissue repair [6].

Postoperative care, therefore, relies extensively on anti-inflammatory pharmacotherapy, particularly non-steroidal anti-inflammatory drugs (NSAIDs) and glucocorticoids. These agents mitigate inflammation by inhibiting the synthesis or activity of proinflammatory effectors or by suppressing arachidonic acid production. Moreover, these drugs can be administered in different forms, including oral treatment, suppository, inhalation, infusion, and local application [7]. However, despite their clinical utility, these drugs remain associated with several adverse effects, such as upper gastrointestinal complications, hypertension, hyperglycemia, psychiatric syndromes, glaucoma, and increased susceptibility to infection. These drawbacks underline the need for safer anti-inflammatory strategies [1].

Carprofen (CP), a non-steroidal anti-inflammatory, also referred to as 2-(6-chloro-9H-carbazol-2-yl) propanoic acid according to IUPAC nomenclature, demonstrates strong fever-reducing, anti-inflammatory, and pain-relieving effects [8,9]. Its pharmacological properties are largely attributed to its carbazole nucleus, a crucial pharmacophore that enables efficient inhibition of the cyclooxygenase (COX) enzyme, which converts arachidonic acid into prostaglandins, including thromboxane and prostacyclin [10,11,12]. Compared with other NSAIDs, the anti-inflammatory potency of CP is similar to piroxicam and diclofenac, and even superior to ibuprofen or phenylbutazone. Currently, CP is not used in humans due to the renal and hepatic toxicity associated with systemic exposure, but it is widely used in veterinary medicine to treat osteoarthritis and bovine mastitis and is advised following soft-tissue injuries or surgical operations to assist in controlling pain and reducing inflammation [13].

Topical or localized delivery may enable harnessing the therapeutic potential of CP while minimizing systemic toxicity, particularly that related to the gastrointestinal tract and kidneys [14]. Local drug delivery in veterinary medicine aims to deliver the minimum amount of the active compound directly to affected animal tissues for a suitable duration. This target approach helps to minimize systemic side effects, reduces the metabolic breakdown of drugs, and reduces dosing frequency [7,15]. In the case of NSADIs, effects on gastric acid and gastrointestinal mucus on the stomach are avoided, and localized delivery systems promote off-target protective effects by preventing therapeutic effects in undesired locations [16].

Nanomedicine has emerged as a promising field for the management of pain and inflammation by enhancing drug efficacy, safety, and localization. Among nanotechnological approaches, nanoemulsions (NEs), also referred to as nanometric-sized emulsions, water-in-oil (w/o) and oil-in-water (o/w) dispersions of two immiscible fluids, represent potential drug delivery systems for topical and localized administration due to their kinetic stability, small droplet size, unique structural, chemical, and mechanical properties, and favorable interaction with skin structures [17]. NEs are indeed highly favored for transdermal drug delivery, particularly in models using porcine skin, which closely mimics human skin permeability. Their effectiveness is derived from a combination of unique structural and physicochemical properties [18]. NEs are preferred for skin drug delivery due to their rapid skin–cell interaction, facilitated by their fluidity and surfactant-stabilized structure, as well as their biphasic structure that enhances the solubility of compounds, protects drugs from degradation, and promotes targeted delivery to inflamed tissues [12,19,20,21,22].

Despite the extensive research on nanoformulations of NSAIDs, the successful incorporation of these drugs into nanocarrier systems remains highly dependent on their physicochemical properties and therapeutic profile, making the development of each formulation drug-specific rather than universally transferable. Therefore, the present study aimed to develop a carprofen-loaded nanoemulsion (CP-NE), perform its physicochemical and biopharmaceutical characterization, and evaluate its therapeutic potential as a topical delivery system for the management of postoperative inflammation in veterinary medicine.

## 2. Materials and Methods

### 2.1. Materials

The CP USP was kindly donated by NGL Fine Chem Ltd. (Mumbai, India), Lauroglycol^®^ 90, Plurol^®^ oleique CC 497, Labrafil^®^ M1944 CS, Labrasol^®^, and Transcutol^®^-P were supplied by Gattefossé (Saint-Priest, France). Castor oil and Tween^®^ 80 were purchased from Sigma-Aldrich (Madrid, Spain). Analytical-grade components were obtained from Panreac (Barcelona, Spain). Water used for all experiments was obtained from a Millipore Milli-Q purification system (Millipore Corporation, Burlington, MA, USA).

### 2.2. Quantification of CP

CP was quantified by high-performance liquid chromatography (HPLC) and UV spectrophotometry. The HPLC analysis was carried out using a Waters 1525 HPLC system with a UV-VIS 2487 detector (Waters, Milford, MA, USA). The Empower Pro software (Waters, Milford, MA, USA) was used to collect and process the data. The assay was performed using a Phenomenex Luna^®^ 5 µm C18 100 Å (150 × 4.6 mm) chromatographic column. The mobile phase consisted of a methanol/phosphate buffer (pH 3; 77:23, *v*/*v*) mixture, which was filtered through a 0.45 µm polyvinylidene fluoride membrane filter (Millipore Corp., Madrid, Spain). The mobile phase was pumped at a flow rate of 1 mL/min. A volume of 10 µL was injected, and the elute was analyzed at 235 nm.

On the other hand, CP quantification by UV spectrophotometry was performed using a Thermo Spectronic Helios Beta UV–Visible Spectrophotometer (Thermo Fisher Scientific, Karlsruhe, Germany). A calibration curve of CP was plotted in the concentration range of 3.125–200 µg/mL using methanol: water (1:1) as solvent, and the drug was determined at 300 nm.

### 2.3. Solubility Studies

The solubility of CP was studied in different oils, including castor oil, Lauroglycol^®^ 90, Plurol^®^ oleique CC 497, and Labrafil^®^ M1944 CS; surfactants such as Labrasol^®^ and Tween^®^ 80; and cosurfactants such as Transcutol^®^-P and isostearyl isostearate. For this experiment, an excess of drug was added to 2 g of these vehicles, and the mixture was stirred at 25 °C for 4 h. The samples were equilibrated overnight and then centrifuged at 9000 rpm for 20 min. The supernatant was diluted with methanol: water (50:50), and the amount of dissolved drug was quantified by UV spectrophotometry at 300 nm using a Helios Beta Spectrophotometer (Thermo Scientific, Barcelona, Spain).

### 2.4. Pseudo-Ternary Phase Diagrams

Five pseudo-ternary phase diagrams were designed by the water titration method using Plurol oleique CC 497 as the oil phase, a mixture of Labrasol^®^ and Transcutol^®^-P at different ratios (S_mix_: 1:1, 1:2, 2:1, 3:1, and 4:1) as surfactant and cosurfactant, respectively, and purified water as the aqueous phase. Oil and S_mix_ were mixed at ratios from 9:1 to 1:9 (*w*/*w*) for 5 min, and then water was added by titration until turbidity or phase separation was achieved to establish the emulsification area of each diagram. Among these pseudo-ternary phase diagrams, the one with the largest emulsification area was selected as the optimal S_mix_ ratio.

### 2.5. Preparation of CP-NE

For the CP-NE (10 mg/g) preparation, the drug was dissolved in the oil phase by stirring at 700 rpm for 10 min. Thereafter, the surfactant and cosurfactant were incorporated by stirring for an additional 10 min. Finally, the aqueous phase was added, maintaining the same stirring conditions until a translucent, homogeneous NE was obtained.

### 2.6. Physicochemical Characterization of CP-NE

The pH of CP-NE was determined at room temperature using a calibrated digital pH meter GLP 22 (Crison Instruments; Barcelona, Spain).

Droplet size, polydispersity index (PI), and zeta potential values of CP-NE were measured in triplicate by dynamic light scattering (DLS) using a Zetasizer Nano ZS (Malvern Instruments, Malvern, UK) at 25 °C, and the formulation without dilution after 1 day of preparation.

The morphology of CP-NE was examined by transmission electron microscopy (TEM) using a JEOL JEM-1010 electron microscope (JEOL Ltd.; Tokyo, Japan). An undiluted sample of the formulation was analyzed by negative staining with uranyl acetate and 24 h of drying.

The viscosity and rheological behavior of the CP-NE formulation were characterized 24 h after preparation using a HAAKE RheoStress 1 rotational rheometer (Thermo Fisher Scientific, Karlsruhe, Germany). Measurements were conducted under a controlled three-step shear protocol consisting of an initial shear rate ramp-up from 0 to 50 s^−1^ over 3 min, followed by a constant shear phase at 50 s^−1^ for 1 min, and a final shear rate ramp-down from 50 to 0 s^−1^ over 3 min. The obtained flow curve data were subsequently fitted to different rheological models (Newton, Bingham, Ostwald-de-Waele, Casson, Herschel-Bulkley, and Cross) to identify the model that best described the experimental behavior, based on the statistical correlation coefficient value (r).

The spreadability of CP-NE was evaluated in triplicate using 0.10 g of the nanoemulsion, which was placed at the center of a glass base plate, and a plastic sheet weighing 3 g was carefully positioned over the sample. After allowing the system to equilibrate for 1 min, the area covered by the formulation was measured. Additional weights (4, 5, 8, 13, and 23 g) were then sequentially applied at 30 s intervals, and the corresponding spreading area was recorded after each load.

### 2.7. Microbiological Quality Control

Microbiological quality control was performed on both the CP-NE and the blank nanoemulsion (Blank-NE). A 1:10 initial dilution of each formulation was prepared in sterile peptone water, followed by serial decimal dilutions (10^−1^, 10^−2^, and 10^−3^). Subsequently, 100 μL of each dilution was plated in triplicate (*n* = 3) onto the appropriate culture media. Sabouraud dextrose agar (SDA) was used for the detection of fungi and yeasts, standard method agar (SMA) for total aerobic mesophilic microorganisms, and Violet Red Bile Agar (VRBA) for Gram-negative bacteria. Additionally, 5% sheep blood agar plates were employed to assess the potential presence of *Staphylococcus aureus* and *Pseudomonas aeruginosa*. SMA, VRBA, and blood agar plates were incubated at 37 ± 0.5 °C for 24–48 h in a Thermo Scientific Heratherm incubator (Waltham, MA, USA), while SDA plates were incubated at 25 ± 1 °C for 5 days. After the respective incubation periods, colonies were enumerated and reported as colony-forming units per milliliter (CFU/mL) of formulation.

### 2.8. Stability Studies

The developed CP-NE was subjected to both physical and chemical stability studies. Physical stability was assessed by multiple light scattering using the TurbiScan Lab technology (Formulation, L’Union, France). Transmission (T) profiles of 20 mL samples of CP-NE stored at 25 °C were recorded on days 1 and 60 to detect destabilization phenomena such as droplet aggregation, coalescence, or migration. Chemical stability was evaluated by quantifying CP content in samples stored at 4 °C, 25 °C, and 40 °C for 40 days. Drug content was determined at predetermined time points (0, 7, 15, and 40 days) to assess possible degradation under different temperature conditions.

### 2.9. In Vitro Release Study

The release experiment of CP from the developed nanoemulsion was carried out using Franz diffusion cells of 14 mL and an effective diffusion area of 2.54 cm^2^ (FDC 400; Crown Grass; Somerville, NJ, USA). These diffusion cells were filled with receptor solution composed of water and 5% Transcutol-P. The system was maintained at 32 °C and continuously stirred to achieve sink conditions. A dialysis membrane (MWCO 12 KDa) previously hydrated with methanol: water (1:1) for 24 h was mounted between the donor and receptor compartments. A volume of 200 µL of CP-NE was placed in the donor compartment, and then 300 µL of sample was extracted from the receptor compartment and replaced with the same volume of receptor medium at predetermined time intervals. These aliquots were analyzed by HPLC, and the results were analyzed as mean ± SD from five replicates. The amount of drug released was plotted as a function of time, and the data were fitted to the following kinetic models: first-order, Higuchi, Exponential growth, Hyperbolic, Weibull, and Korsmeyer–Peppas. The model with the best fit of the experimental data was selected based on the coefficient of determination (r^2^) and Akaike Information Criterion (AIC).

### 2.10. Ex Vivo Permeation Study

Ex vivo permeation studies were performed using pig ear skin obtained from the Animal Facility of the Faculty of Medicine, in accordance with the Animal Experimentation Ethical Committee of the University of Barcelona, Spain (CEEA-UB), with reference number N°001, dated 10 January 2019. Skin integrity was evaluated by determining transepidermal water loss (TEWL) with a Tewameter TM 300 (Courage & Khazaka Electronics GmbH, Cologne, Germany), and only those samples with TEWL readings under 10 g/m^2^·h were included in the study. Skin membranes (0.7 mm thickness) were mounted in Franz diffusion cells with a capacity of 6 mL and a diffusion area of 0.64 cm^2^. The receptor phase consisted of buffer solution pH 7.4 and 5% Transcutol^®^-P. The system was maintained at 32 °C and stirred at 600 rpm to ensure sink conditions. CP-NE (200 µL) was added to the donor compartment, and at specific time points over a 24 h period, 300 µL samples were withdrawn from the receptor compartment and replaced with an equal volume of receptor medium. The collected samples were analyzed using HPLC to determine the amount of permeated drug.

At the end of the permeation experiments, the skin samples were removed from the Franz diffusion cells, rinsed with distilled water, and trimmed to isolate the exposed permeation area. The resulting tissue was weighed, and the CP retained within the skin (Q_ret_, µg/cm^2^) was determined by ultrasound-assisted extraction using 2 mL of methanol, followed by sonication for 20 min. The extracts were then filtered and quantified by HPLC analysis.

An additional biopharmaceutical assessment was conducted to calculate several permeation and predictive parameters, including steady-state flux (J, μg/h), permeability coefficient (K_p_, cm/h), lag time (Tl, h), vehicle-to-tissue partition coefficient (P_1_, cm), diffusion coefficient (P_2_, 1/h), and the estimated steady-state plasma concentration (C_ss_, µg/mL) [23].

### 2.11. Safety and Biocompatibility Evaluation

#### 2.11.1. Cytotoxicity Assay

The cytotoxic effects of CP-NE, Blank-NE, and free drug were evaluated using an MTT (3-(4,5-dimethylthiazol-2-yl)-2,5-diphenyltetrazolium bromide) assay. The HaCaT cell line, which consists of immortalized human keratinocytes, was plated at a density of 2 × 10^5^ cells/mL in Corning 96-well plates (Thermo Fisher Scientific, Waltham, MA, USA) and incubated at 37 °C in a humidified environment with 5% CO_2_ for 24 h to facilitate cell attachment. Experiments were performed once the cells reached approximately 80–90% confluence. HaCaT cells were cultured in Dulbecco’s Modified Eagle Medium (DMEM) with high glucose content, supplemented with 25 mM HEPES, 1% non-essential amino acids, 100 U/mL penicillin, 100 mg/mL streptomycin, and 10% heat-inactivated Fetal Bovine Serum (FBS). Different concentrations of CP-NE, Blank-NE, and free CP were tested (1, 0.1, 0.01, 0.001, 0.0001, 0.005, 0.0025, 0.00125, 0.000625, 0.0003125 mg/mL). After 24 h of exposure, the cells were washed with sterile phosphate-buffered saline (PBS) and incubated with MTT solution (5 mg/mL) for 2 h at 37 °C. Following incubation, the culture medium was carefully removed, and 0.1 mL of dimethyl sulfoxide (DMSO, 99% purity) was added to lyse the cells and dissolve the purple MTT crystals. The resulting cell lysate was transferred to a fresh 96-well plate, and absorbance was measured at 540 nm (excitation) and 630 nm (emission) using an Automatic Microplate Reader (Modulus Microplate Multimode Reader, Turner Biosystems, Sunnyvale, CA, USA). Untreated cells were used as the negative control. The absorbance values were directly proportional to cell viability, and the percentage of viable cells was calculated using Equation (1):

(1) $$Cell\;viability=\;\frac{ABS\;treated\;cell}{ABS\;control\;cells}\;\times 100$$

#### 2.11.2. Ex Vivo Skin Toxicity

Pig ear skin samples were exposed for 24 h to physiological saline, CP-free solution, CP-NE, and Blank-NE in the receptor compartment of Franz diffusion cells. After the exposure period, the skin samples were carefully removed, rinsed with sterile distilled water to eliminate residual formulation, and fixed in a suitable fixative solution for subsequent histological analysis (Section 2.12.2). This analysis was performed to evaluate potential morphological alterations and assess the compatibility of the tested formulations with skin tissue.

#### 2.11.3. Skin Tolerance and Biomechanical Evaluation

The biomechanical properties of the skin, including Transepidermal Water Loss (TEWL) and Stratum Corneum Hydration (SCH), were evaluated before and after the topical application of CP-NE on the previously shaved dorsal skin of CD-1 male mice (n = 5). Before measurements, the animals were acclimatized in a controlled environment (25 °C and 53.7% relative humidity) for approximately 30 min. TEWL and SCH values were measured before formulation application and at 0.5, 1.5, 2.5, 4, and 8 h after the application of CP-NE. TEWL values were measured with a Tewameter^®^ TM 300 (Courage-Khazaka electronic GmbH, Cologne, Germany) and stratum corneum hydration (SCH) with a Corneometer^®^ CM 825 (Courage-Khazaka electronic GmbH). This study was approved by the Animal Ethics Committee of the National Autonomous University of Honduras (UNAH) under protocol code CICUAL-002-2025.

#### 2.11.4. In Vitro HET-CAM Assay

The irritation potential of CP-NE was evaluated using the Hen’s Egg Test on the Chorioallantoic Membrane (HET-CAM). Fertilized chicken eggs (10 days of incubation) obtained from a commercial supplier (G.A.L.L.S.A. farm, Tarragona, Spain) were used for the assay. The eggshell and the underlying inner membrane were carefully removed to expose the chorioallantoic membrane (CAM), which separates the embryo from the air chamber. A volume of 0.3 mL of CP-NE and Blank-NE was gently applied onto the CAM surface. A 0.9% sodium chloride solution was used as the negative control, while a 0.1 N sodium hydroxide solution served as the positive control. After application, the membranes were observed for 5 min to detect potential irritation responses, including hemorrhage, vascular lysis, and coagulation. The irritation score (IS) was calculated according to Equation (2):

(2) $$IS=\left[\frac{\left(301-hemorrhage\;time\right)}{300}\times 5\right]+\left[\frac{\left(301-lysis\;time\right)}{300}\times 7\right]+\left[\frac{\left(301-coagulation\;time\right)}{300}\times 9\right]$$

The irritation potential of the samples was classified according to the following scale:

IS between 0 and 0.99: non-irritant; IS between 1.0 and 4.99: slightly irritant; IS between 5.0 and 9.99: moderately irritant; and IS between 10.0 and 21.0: extremely irritant.

### 2.12. Efficacy Studies

#### 2.12.1. Animals and Study Protocol

To evaluate the efficacy of CP-NE in the management of post-surgical inflammation, four groups of male CD-1 mice (n = 6 per group) were established. All experimental procedures were approved by the Animal Ethics Committee of the National Autonomous University of Honduras (UNAH) under protocol code CICUAL-002-2025. The animals were anesthetized with ketamine (Ketonal^®^ 100) and xylazine hydrochloride (Xilapet^®^ 2%) at doses adjusted according to body weight. The dorsal area of each mouse was carefully shaved prior to the surgical procedure. A skin incision of approximately 1.5–2 cm was then made using a sterile scalpel, and the wound was subsequently sutured. The experimental conditions for each study group are described in Table 1. CP-NE was administered topically twice daily (every 12 h) for 7 consecutive days. At the end of the treatment period, the animals were euthanized in a CO_2_ chamber. The dorsal skin samples were excised, rinsed with sterile distilled water, and processed for subsequent histological analysis.

#### 2.12.2. Histological Analysis

The mouse skin samples were fixed for 24 h in Orth-ER fixative solution, protected from light and heat. After fixation, the excess fixative was removed by washing the samples with water for 4 h. The tissues were subsequently dehydrated through a graded ethanol series (50%, 60%, 70%, 80%, 90%, 95%, and 99%) and then cleared with xylene (DIMELAB, Tegucigalpa, Honduras). The processed tissues were sectioned at a thickness of 10 μm using a Minot-type microtome (American Optical Co., Buffalo, NY, USA). The obtained sections were mounted onto microscope slides previously coated with an adhesive solution (Haupt’s adhesive). The tissue sections were then stained with hematoxylin and eosin (Merck, Darmstadt, Germany). Finally, the slides were mounted using Entellan^®^ resin (Merck, Darmstadt, Germany), dried in an oven at 28.0 ± 1.0 °C, and allowed to stabilize for at least 24 h. The histological samples were subsequently observed and analyzed using an Olympus CX31 microscope equipped with a digital camera.

A blinded, semi-quantitative histopathological analysis was performed to compare the inflammatory response and tissue healing across the experimental groups. Three representative samples from each group, including positive control, negative control, diclofenac-treated, blank nanoemulsion-treated, and CP-NE animals, were evaluated independently by two blinded analysts without prior knowledge of treatment allocation. The evaluated histopathological parameters included dermal inflammatory infiltrate, epidermal infiltrate, dermal edema, epidermal thickening, and dermal tissue damage. Each parameter was scored on an ordinal scale based on the severity of the histological findings: 0 = absent, 1 = mild, 2 = moderate, and 3 = severe. The final score for each sample was calculated as the average of the scores assigned by both evaluators. This semi-quantitative scoring approach was used to provide a comparative assessment of tissue inflammation and wound-healing progression across the different treatment groups.

### 2.13. Statistical Analysis

Statistical analyses were carried out using GraphPad Prism version 5.0 software (GraphPad Software Inc., San Diego, CA, USA). Data are expressed as mean ± standard deviation (SD) from three independent experiments (n = 3). Comparisons among groups were performed using one-way analysis of variance (ANOVA) followed by Tukey’s post hoc test. Differences were considered statistically significant when *p*-values were lower than 0.05.

## 3. Results

### 3.1. Solubility Studies

**Figure 1:**
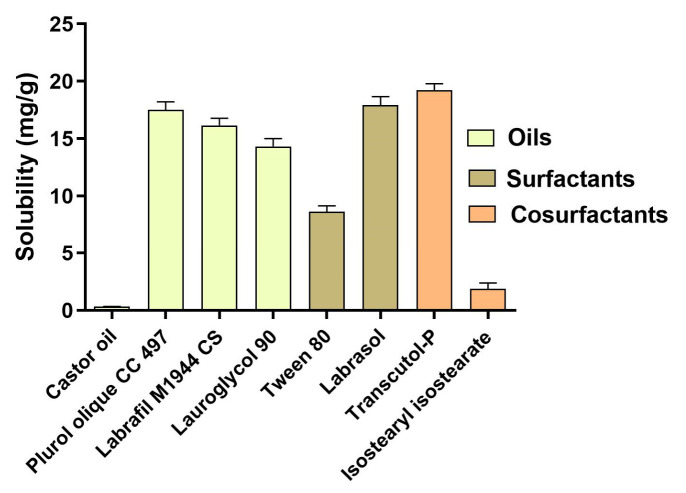
Carprofen solubility in different oils, surfactants, and cosurfactants. Results are given as mean ± SD (n = 3).

Figure 1 shows the solubility of CP in different excipients. Based on these results, Plurol^®^ oleique CC 497 was selected as the oil phase, Labrasol^®^ as the surfactant, and Transcutol^®^-P as the cosurfactant, as these components showed the greatest potential for drug solubilization.

### 3.2. Pseudo-Ternary Phase Diagrams and CP-NE Composition

**Figure 2:**
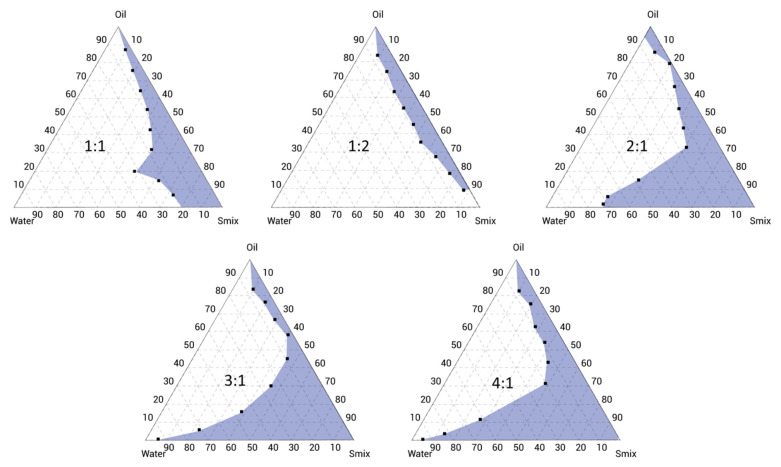
Pseudo-Ternary Phase Diagrams at different ratios of Labrasol^®^/Transcutol^®^-P (S_mix_ 1:1, 1:2, 2:1, 3:1, and 4:1).

Five pseudo-ternary phase diagrams were constructed using Plurol^®^ oleique CC 497 as the oil phase, Labrasol^®^ as the surfactant, and Transcutol^®^-P as the co-surfactant (Figure 2). The largest area of NE formation was obtained with a S_mix_ of Labrasol^®^/Transcutol^®^-P at the ratio of 4:1, and thus it was selected as the optimized S_mix_ ratio for CP-NE formulation.

Table 2 presents the composition of CP-NE, which was formulated by incorporating CP (10 mg/g) into 10% Plurol^®^ oleique CC 497 as the oil phase, 28% Labrasol^®^ as the surfactant, 7% Transcutol^®^-P as the cosurfactant, and 55% purified water. This final formulation showed a translucent, monophasic, homogeneous appearance and was free of visible particles or precipitates.

### 3.3. Characterization of CP-NE

**Figure 3:**
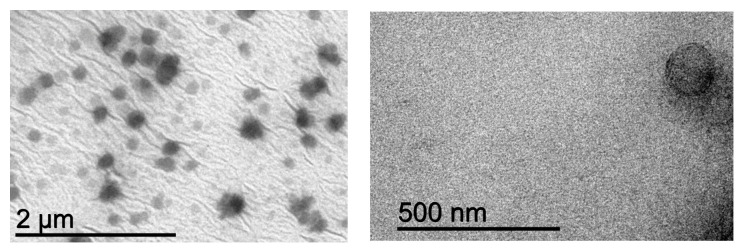
Morphology analysis of carprofen nanoemulsion (CP-NE) by Transmission electron microscopy (TEM). Magnification 25,000× and 80,000×.

The developed CP-NE exhibited a homogeneous, transparent appearance, with no evidence of phase separation, drug precipitation, or particulate matter, indicating good physical integrity of the formulation. The pH of the CP-NE was 5.5, which is considered suitable for dermal applications and compatible with the skin’s physiological pH. DLS analysis revealed a mean droplet size of 140.06 ± 3.85 nm with a polydispersity index (PDI) of 0.301 ± 0.08, suggesting a narrow size distribution and adequate homogeneity of the NE system. The droplet size determined by DLS was further corroborated by transmission electron microscopy (TEM), which revealed well-defined, spherical droplets with sizes consistent with those obtained by DLS analysis (Figure 3).

**Figure 4:**
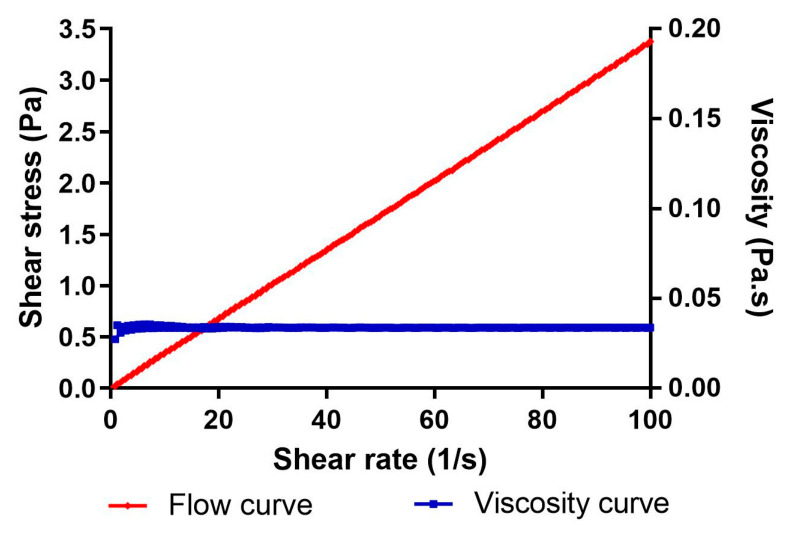
Rheological profile of carprofen nanoemulsion (CP-NE) at 25 °C showing both flow and viscosity curves.

As shown in Figure 4, the flow curve of the CP-NE formulation exhibited a linear relationship between shear stress and shear rate over the evaluated range. Accordingly, the apparent viscosity remained constant throughout the shear rate interval, showing a value of 33.71 mPa·s. This behavior is characteristic of a Newtonian fluid, as confirmed by mathematical modeling of the experimental data, which showed that the Newton model provided the best fit, with a correlation coefficient (r) of 1.

**Figure 5:**
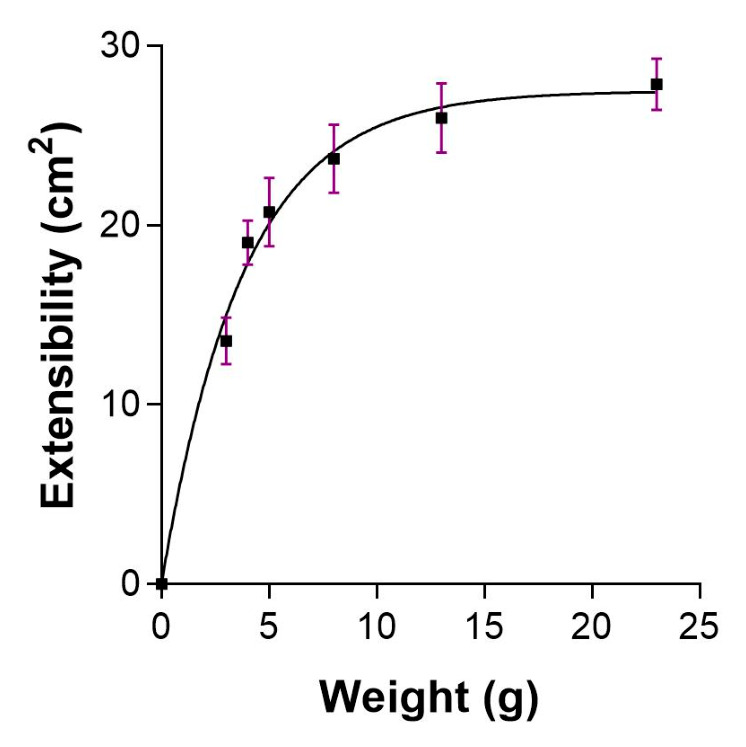
Spreadability profile of carprofen nanoemulsion (CP-NE) at 25 °C. Results are given as mean ± SD (n = 3).

The spreadability assay showed that CP-NE extended progressively as increasing weights were applied (Figure 5). The formulation spread easily across the surface, reaching a maximum spreading area of approximately 28 cm^2^ under the highest applied load. Mathematical modeling of the experimental data indicated that the spreading behavior followed a one-phase exponential association profile, with a high coefficient of determination (r^2^ = 0.97).

### 3.4. Microbiological Quality Control

**Figure 6:**
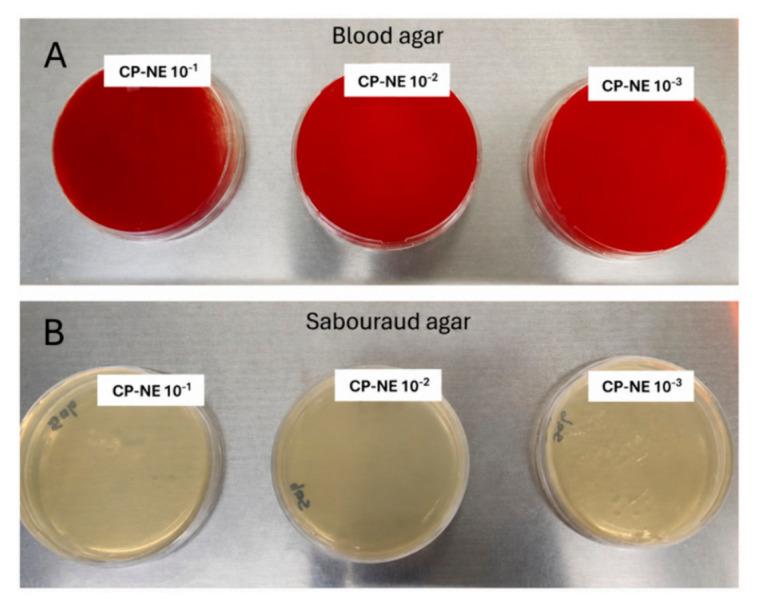
Microbiological quality control of carprofen nanoemulsion (CP-NE). (**A**) Seeding in blood agar and (**B**) seeding in Sabouraud agar of three different concentrations of CP-NE.

No microbial growth was detected in CP-NE or Blank-NE samples on Sabouraud dextrose agar (SDA), standard method agar (SMA), or Violet Red Bile Agar (VRBA) plates after the respective incubation periods. Additionally, no growth of *Staphylococcus aureus* or *Pseudomonas aeruginosa* was observed on 5% blood agar plates, with microbial counts below the detection limit (<10 CFU/mL). These findings indicate that both formulations complied with microbiological quality requirements and were free from detectable contamination under the tested conditions, supporting their suitability for topical skin application (Figure 6).

### 3.5. Physical and Chemical Stability

**Figure 7:**
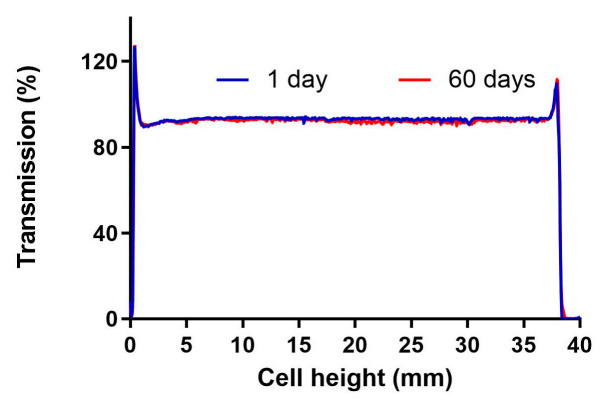
Transmission profiles of carprofen nanoemulsion (CP-NE) over the 60-day storage period at 25 °C.

Variations greater than 10% in the transmission signals would indicate destabilization phenomena. The peaks observed on the left and right sides of the profiles correspond to the meniscus formed at the interface between the sample and the glass cell. As shown in Figure 7, the transmission profiles (%) of CP-NE remained constant under the studied conditions, confirming that the formulation is physically stable and does not exhibit signs of flocculation, sedimentation, or coalescence over the 60-day storage period.

**Figure 8:**
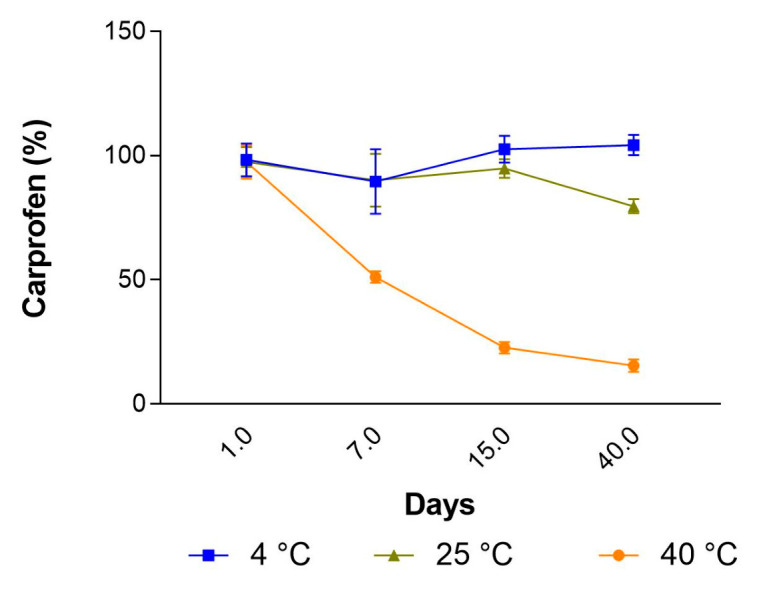
Chemical stability of carprofen nanoemulsion (CP-NE) over a period of 40 days at 4 °C, 25 °C, and 40 °C. Results are given as mean ± SD (n = 3).

The chemical stability of CP incorporated into the nanoemulsion was evaluated over a period of 40 days under different storage conditions (4 °C, 25 °C, and 40 °C). As shown in Figure 8, the CP-NE stored at 4 °C exhibited no detectable drug degradation over the study period, indicating excellent chemical stability under refrigerated conditions. At 25 °C, a slight decrease in CP content was observed after 40 days; however, this reduction was not statistically significant, suggesting that the formulation maintains an acceptable level of chemical stability at room temperature. In contrast, storage at 40 °C resulted in a significant degradation of CP, evidenced by a marked reduction in drug content compared to the initial value.

### 3.6. In Vitro Release Study

**Figure 9:**
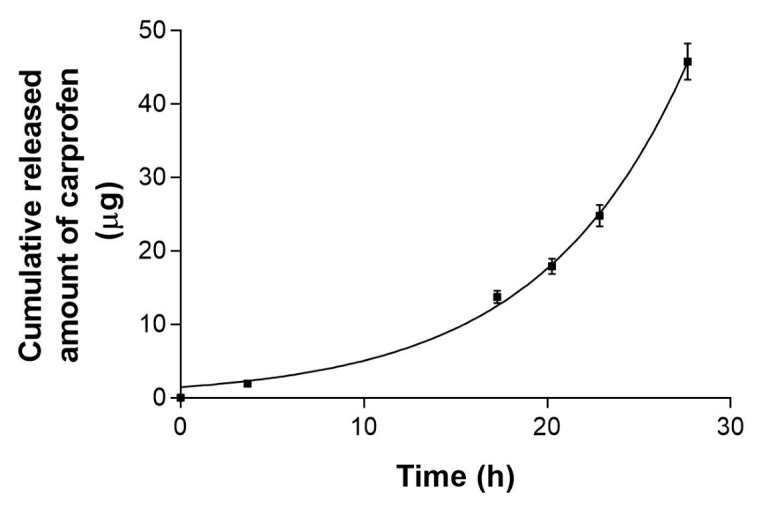
In vitro release profile of carprofen from nanoemulsion. The results are presented as mean ± SD (n = 5).

At the end of the experiment, an amount of 45.77 µg of CP was released (Figure 9). The drug release follows an exponential growth kinetics with a r^2^ of 0.99 and a lower AIC of 5.53, which increases exponentially with a rate constant (K) of 0.1247 h^−1^. This release profile shows that drug release accelerates over time and has a half-life of 5.6 h.

### 3.7. Ex Vivo Permeation Studies

**Figure 10:**
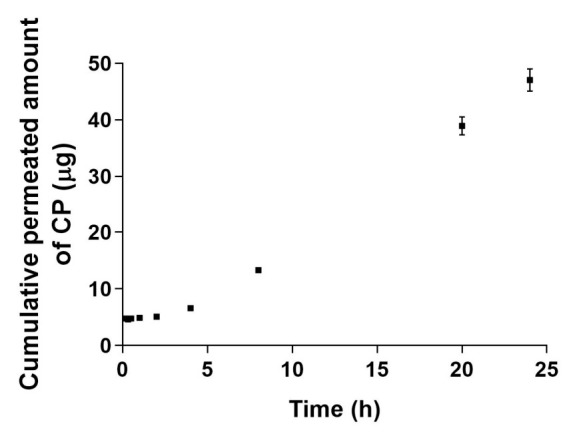
Cumulative permeated amount of carprofen from nanoemulsion. The results are presented as mean ± SD (n = 6).

Ex vivo permeation studies showed that only a minimal amount of drug is able to penetrate the skin after 24 h of assay. Figure 10 shows the linear segment obtained from the ex vivo permeation profile of CP-NE through porcine ear skin, which was used to carry out further biopharmaceutical studies.

The results of the permeation and prediction parameters of CP-NE (Table 3) revealed that only a minimal portion of the CP incorporated into the nanoemulsion could diffuse and penetrate the skin with a transdermal flow of 2.052 µg/h/cm^2^, and a permeability coefficient (K_p_) of 2.052 × 10^−4^ cm/h. The vehicle/tissue partition coefficient (P_1_) and the diffusion coefficient (P_2_) were 1.35 × 10^−3^ cm and 0.152 1/h, respectively. The time taken for CP to appear in systemic circulation was 1.096 h. The predicted amount of drug that would reach a systemic level upon reaching steady state (C_ss_) was 0.040 µg/mL, considering an area of application of 5 cm^2^ and a plasma clearance of the drug of 0.255 L/h. Finally, the amount of CP retained in the tissue (Q_ret_) was 791.02 µg/g skin/cm^2^.

### 3.8. Safety and Biocompatibility Evaluation

#### 3.8.1. Cytotoxicity Assay

**Figure 11:**
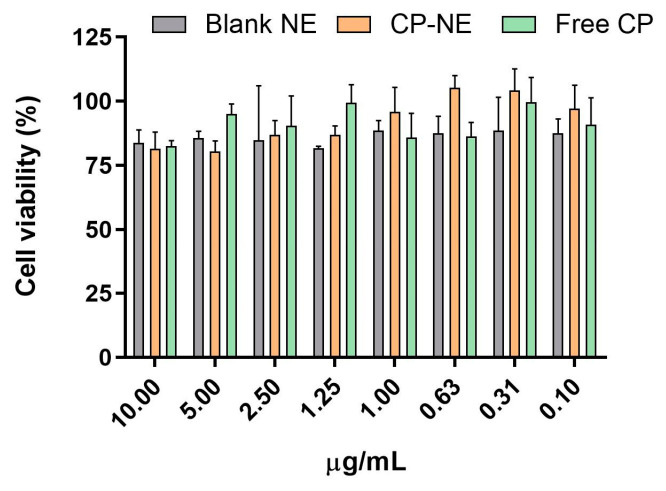
Citotoxicity assay of blank-NE, CP-NE, and Free CP on HaCaT keratinocyte cells (n = 3).

The cytotoxicity of the CP-NE was evaluated in HaCaT keratinocyte cells using the MTT assay and compared with the free CP and the Blank-NE. As shown in Figure 11, cell viability remained above 80% for all tested concentrations, ranging from 10 to 0.10 µg/mL, for CP-NE, free CP, and the Blank-NE. No statistically significant differences in cell viability were observed among the three groups across the evaluated concentration range. These results indicate that neither the nanoemulsion components nor the incorporation of CP induced cytotoxic effects in keratinocytes under the experimental conditions employed. According to these results, CP-NE formulation can be regarded as biocompatible and suitable for topical application.

#### 3.8.2. Ex Vivo Skin Toxicity

**Figure 12:**
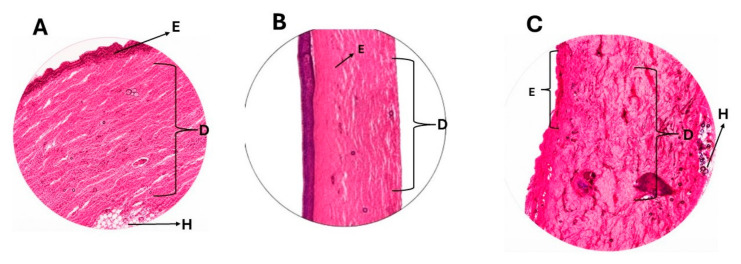
Histological representations of ex vivo toxicity in pig skin. (**A**) Negative control treated with physiological saline, (**B**) Porcine skin in contact with Blank-NE, and (**C**) Porcine skin in contact with CP-NE. E: epidermis, D: dermis, H: hypodermis.

The histological analysis of pig skin samples exposed to CP-NE (Figure 12C) and Blank-NE (Figure 12B) revealed no structural alterations compared with the negative control treated with physiological saline (Figure 12A). As can be observed, the epidermal and dermal architecture remained intact in all samples, with no evidence of tissue disruption or cellular damage. These findings indicate that neither nanoemulsion nor its excipients induced detectable histological changes in pig skin under the tested conditions.

#### 3.8.3. Skin Tolerance and Biomechanical Evaluation

**Figure 13:**
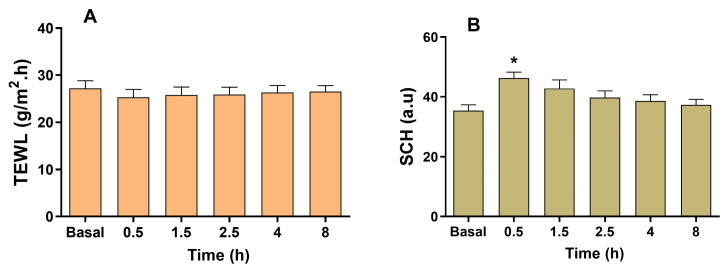
Biomechanical parameters in the baseline state and after the application of carprofen nanoemulsion (CP-NE). (**A**) Transepidermal water loss (TEWL) and (**B**) stratum corneum hydration (SCH). Results are expressed as mean ± SD (n = 3). Significant statistical differences * *p* < 0.05 comparison with the basal state.

Figure 13 shows the biomechanical skin parameters evaluated before and after topical application of CP-NE to the dorsal skin of mice. TEWL values did not show statistically significant differences throughout the 8 h evaluation period following formulation application (*p* > 0.05), indicating that the skin barrier function remained unaltered under the tested conditions. In contrast, a significant increase in SCH values was observed immediately after application (0.5 h). From 1.5 h, SCH values remained slightly elevated compared with baseline levels, although these differences were not statistically significant and gradually tended to return to initial values.

#### 3.8.4. In Vitro HET-CAM Assay

**Figure 14:**
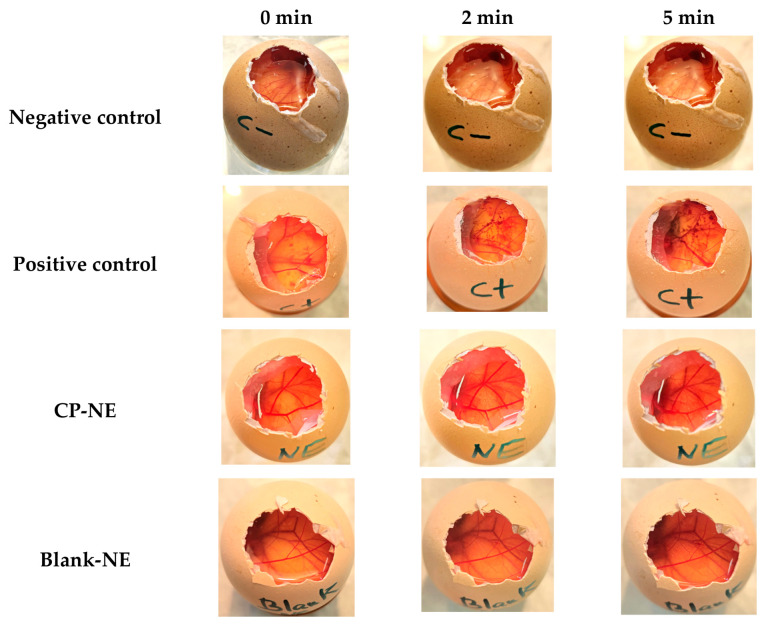
Assessment of the irritation potential by the Hen’s Egg Test on the Chorioallantoic Membrane (HET-CAM). Samples evaluated for 5 min: negative control (saline solution), positive control (0.1 N sodium hydroxide solution), carprofen nanoemulsion (CP-NE), and Blank-NE.

The experiment performed with the embryonated eggs showed that both Blank-NE and CP-NE were slightly irritant, showing an IS of 2.3 ± 0.35 and 2.7 ± 0.28, respectively (Figure 14). Therefore, it is recommended to avoid contact with the ocular mucosa during periocular application.

### 3.9. Efficacy Studies

#### Anti-Inflammatory Efficacy of CP-NE in Mice

**Figure 15:**
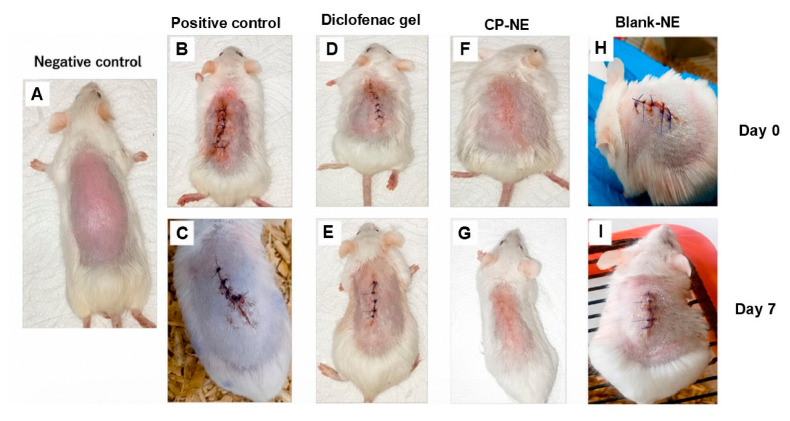
In vivo efficacy evaluation. (**A**) Negative control (day 1), (**B**,**C**) Positive control, (**D**,**E**) diclofenac gel treatment, (**F**,**G**) carprofen nanoemulsion (CP-NE) treatment, (**H**,**I**) Blank-NE treatment.

Figure 15 shows the dorsal skin of mice after the surgical procedure and following 7 days of treatment with CP-NE and diclofenac gel. The negative control group (Figure 15A), in which no incision or treatment was applied, showed normal skin morphology without visible signs of inflammation. In contrast, the positive control group (Figure 15B,C), subjected to the surgical incision but without anti-inflammatory treatment, exhibited evident redness and inflammatory signs in the affected area. Mice treated with diclofenac gel (Figure 15D,E) and CP-NE (Figure 15F,G) showed a marked reduction in redness and inflammation at the end of the treatment period compared with the positive control group. Finally, Blank-NE (Figure 15H,I) did not exhibit an anti-inflammatory activity, confirming that the vehicle itself did not exert a relevant anti-inflammatory effect.

**Figure 16:**
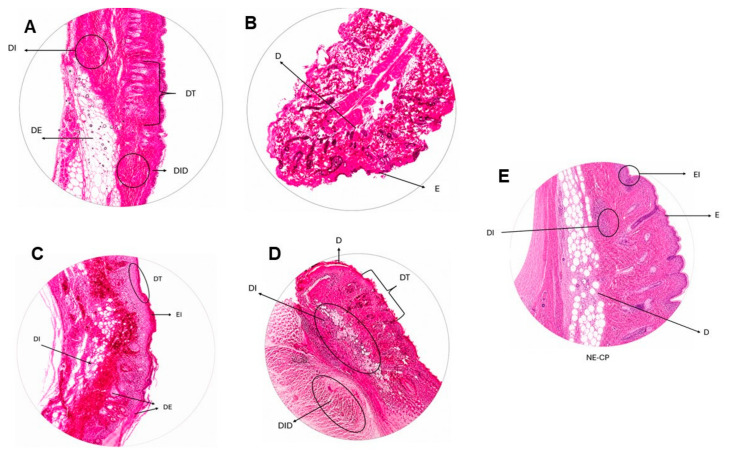
Histological analysis of dorsal skin samples from the in vivo experiment. (**A**) Positive control, (**B**) Negative control, (**C**) Treatment with commercial diclofenac gel, (**D**) Treatment with carprofen nanoemulsion (CP-NE), and (**E**) Blank-NE. E: Epidermis; D: Dermis; DI: dermal infiltrates; DE: dermal edema; DT: Epidermal thickening; DID: Dermal tissue damage.

Figure 16 presents the histological analysis of dorsal skin samples from the mice. In the positive control group (Figure 16A), histological evaluation revealed a moderate inflammatory response, predominantly localized in the dermis, accompanied by moderate edema and partial dermal architectural alteration. The epidermis showed moderate thickening with a mild inflammatory infiltrate. Although tissue organization was altered in certain areas, the overall skin structures remained preserved, indicating moderate tissue damage. In contrast, the negative control group (Figure 16B) showed only mild histological alterations attributable to sample handling, with no evidence of edema or significant tissue damage. The epidermal and dermal architecture remained preserved, suggesting minimal tissue injury and a controlled inflammatory response, consistent with normal skin morphology. Skin treated with diclofenac (Figure 16C) showed a severe, diffuse dermal inflammatory infiltrate, characterized by high cell density, composed predominantly of mononuclear cells that occupied a large portion of the dermal tissue. Involvement of the dermoepidermal junction was also observed, with a moderate inflammatory infiltrate extending into the epidermis. Mild dermal edema and epidermal thickening were also detected. Treatment with Blank-NE (Figure 16E) induced inflammatory infiltrates in both the epidermis and dermis, associated with dermal edema. Epidermal thickening and evidence of dermal tissue damage were also observed. In contrast, skin treated with CP-NE showed only mild inflammatory infiltrates in the epidermis and dermis. Both epidermal and dermal structures appeared preserved and healthy, without evident edema or tissue damage, indicating a favorable histological profile.

Table 4 shows the results of the semi-quantitative, blinded histopathological analysis in which inflammatory parameters were evaluated, including dermal inflammatory infiltrate, epidermal infiltrate, dermal edema, epidermal thickening, and dermal tissue damage. The positive control group showed elevated histopathological scores for most assessed parameters, particularly the dermal inflammatory infiltrate and dermal tissue damage, indicating a persistent inflammatory response and poor tissue recovery. In contrast, the negative control group showed minimal histological alterations. The group treated with diclofenac demonstrated partial histological improvement compared to the positive control group, with reduced scores for epidermal infiltrate and dermal edema. However, moderate dermal inflammatory infiltrate and tissue damage were still observed. It is noteworthy that the group treated with CP-NE exhibited lower scores for dermal inflammatory infiltrate, epidermal thickening, and dermal tissue damage than the group treated with diclofenac, suggesting a better anti-inflammatory effect and improved tissue recovery. In contrast, the group treated with Blank-NE showed high inflammation and tissue damage scores, comparable to those observed in the positive control group, indicating that the therapeutic effect was associated with CP rather than with the nanoemulsion vehicle itself. Overall, the semi-quantitative histopathological analysis corroborated the qualitative microscopic observations and demonstrated a trend toward improved wound healing and a reduced inflammatory response in the animals treated with CP-NE.

## 4. Discussion

The present study arises from the need to provide alternative strategies for the administration of CP, whose renal and hepatic toxicity associated with systemic exposure has limited the clinical exploitation of its potent anti-inflammatory activity. In this context, the development of topical delivery systems represents an attractive approach to take advantage of the therapeutic benefits of this drug while minimizing systemic adverse effects. Accordingly, this work proposes the development and evaluation of a CP-loaded nanoemulsion (CP-NE) as a topical delivery system for the management of post-surgical inflammation in veterinary medicine. By localizing the drug at the site of application, this strategy aims to achieve a predominantly local anti-inflammatory effect while avoiding the systemic exposure responsible for the adverse effects traditionally associated with CP.

CP has previously been incorporated into nanosystems such as PLGA nanoparticles and evaluated for dermal administration to reduce inflammation in animal models [24]. However, its incorporation into a nanoemulsion for topical application has not been widely explored [9]. In the present study, CP-NE was developed using a combination of oils, surfactants, and co-surfactants, including Plurol Oleique^®^, Labrasol^®^, and Transcutol^®^, three excipients widely used in colloidal systems intended for dermal delivery due to their excellent solubilizing capacity and good skin compatibility [25]. Plurol Oleique^®^ acts as a solubilizer and bioavailability enhancer for poorly soluble drugs. In combination with Capriol 90 (1:1), this excipient has previously been selected as the oil phase in self-emulsifying drug delivery systems [26]. Labrasol^®^ is a nonionic oil-in-water surfactant composed of mixtures of mono-, di-, and triglycerides, as well as mono- and di-fatty acid esters of polyethylene glycol, and is commonly used as a solubilizer, stabilizer, and permeation enhancer in pharmaceutical formulations [27]. Transcutol^®^ (diethylene glycol monoethyl ether) is a liquid solvent belonging to the class of hydroalcoholic vehicles and is widely used in human and veterinary medicines administered by different routes, particularly dermal and transdermal routes [28].

The CP-NE developed in this study exhibited a mean droplet size of 140.06 nm, which falls within the typical size range reported for nanoemulsion systems, generally below 200 nm. Such small droplet sizes increase the interfacial surface area upon skin application, facilitating interactions between the formulation components and the stratum corneum and potentially enhancing the cutaneous bioavailability of poorly water-soluble drugs. In addition, a low polydispersity index (PDI) of 0.301 was obtained, indicating a relatively narrow droplet size distribution and good homogeneity of the system. PDI values close to or below 0.3 are commonly associated with physically stable and uniform colloidal dispersions, suggesting an adequate optimization of the surfactant system used in the formulation [29]. Morphological analysis by transmission electron microscopy confirmed the presence of spherical droplets with sizes consistent with those obtained by dynamic light scattering (DLS). Furthermore, the pH of CP-NE remained within the range of 4–5, which is compatible with the physiological pH of the skin (approximately 4.5–5.5). Maintaining a pH within this range is important to preserve the barrier function of the stratum corneum and to minimize the risk of irritation during repeated topical application [30,31].

Rheological properties are closely related to sensory characteristics, dosing behavior, and spreadability, and may also influence key biopharmaceutical parameters such as drug release and skin permeation [32]. In this study, CP-NE exhibited Newtonian flow behavior, characterized by a linear relationship between shear stress and shear rate and a constant viscosity across the evaluated shear rate range. Such behavior is typical of low-viscosity colloidal systems and is advantageous for dermal administration, as it facilitates spreading over the skin surface and promotes uniform distribution of the formulation at the application site, allowing for easy administration by spraying. The relatively low viscosity observed (33.71 mPa·s) may also favor contact between the formulation and the stratum corneum, potentially enhancing drug diffusion across the skin. This rheological profile is consistent with the extensibility results, which showed that the formulation spreads easily over the surface, reaching a maximum spreading area of approximately 28.26 cm^2^ when 100 mg of the formulation was applied. Adequate spreadability is a desirable characteristic for topical systems because it ensures homogeneous coverage of the treated area and improves patient acceptability during application [33].

The absence of detectable microbial growth in CP-NE and Blank-NE indicates that the formulation maintained adequate microbiological quality under the evaluated conditions, and further supports the suitability of the manufacturing and handling procedures used in this study. Ensuring microbiological safety is particularly important for topical formulations, as contamination may compromise product stability and increase the risk of skin irritation or infection, especially when applied to compromised skin such as post-surgical wounds [34].

The stability studies demonstrated that CP-NE maintains adequate physical and chemical integrity under the evaluated storage conditions. The absence of significant variations in the transmission profiles obtained by multiple light scattering indicates that the nanoemulsion remained physically stable over the 60-day period, with no evidence of typical destabilization phenomena such as flocculation, sedimentation, or coalescence. This stability may be attributed to the nanometric droplet size of the system, since the very small droplet size reduces the effect of gravitational forces between dispersed droplets, allowing Brownian motion to dominate the system and contribute to the prevention of creaming or sedimentation over time [35]. In addition, the steric hindrance of the non-ionic surfactant used in the formulation contributes to the stabilization of the oil droplets by forming a protective interfacial layer that limits droplet aggregation [36]. From chemical stability, CP remained stable under refrigerated conditions and showed acceptable stability at room temperature, while storage at 40 °C promoted significant drug degradation. This temperature-dependent behavior is consistent with the susceptibility of many drugs to thermal degradation and highlights the importance of appropriate storage conditions to preserve drug stability [37].

The in vitro release study provided important information regarding the drug delivery performance of the nanoemulsion and its potential in vivo behavior [38]. After 24 h, approximately 11.6 µg/cm^2^ of CP was released from the formulation, considering the tested diffusion area. The release profile followed exponential growth kinetics (r^2^ = 0.99) with a rate constant (K) of 0.1247 h^−1^ and a half-life of 5.6 h, indicating a progressive increase in the release rate over time. Importantly, CP was predominantly retained within the skin (Q_ret_ of 791.02 µg/g skin/cm^2^), while only a minimal amount of 47.50 µg of the drug was able to permeate through the tissue and reach the receptor compartment, representing only 2.38% of the drug initially applied to the donor compartment. These findings suggest that CP-NE may be suitable for achieving a localized therapeutic effect while minimizing systemic exposure and the adverse effects associated with systemic administration. The high amount of CP retained within the skin indicates that the drug was able to successfully penetrate the SC, the main rate-limiting barrier for topical drug delivery, and remain localized within the tissue at potentially effective concentrations in the target area. The ability of CP-NE to facilitate drug penetration across the SC may be attributed to the presence of excipients such as Labrasol^®^, which is known to enhance the solubility and permeation of poorly soluble drugs [27]. Similarly, co-surfactants such as Transcutol^®^ P have been widely reported to act as permeation enhancers by modifying the lipid organization of the stratum corneum and improving drug diffusion through the skin [28]. The predicted systemic exposure associated with CP-NE was extremely low. Based on the permeation parameters obtained, the estimated steady-state plasma concentration (C_ss_) following the application of the formulation to a 5 cm^2^ skin area would be approximately 0.040 µg/mL. This value is far below the plasma concentrations required to produce a systemic anti-inflammatory effect, suggesting that the formulation is unlikely to cause systemic adverse effects. Such a pharmacokinetic profile is particularly desirable for topical anti-inflammatory therapy, as it allows the drug to exert its therapeutic action locally while minimizing systemic exposure. Furthermore, the relatively long lag time (TL = 1.096 h) indicates that during this period, the drug is gradually distributed within the skin layers before reaching systemic circulation. This behavior favors local drug accumulation and contributes to the establishment of a sustained therapeutic effect at the site of application.

The safety and biocompatibility of CP-NE were evaluated through complementary in vitro, ex vivo, and in vivo approaches to ensure that the formulation does not induce adverse effects on the skin. The cytotoxicity assessment performed in HaCaT keratinocytes demonstrated that cell viability remained above 80% across all tested concentrations of CP-NE, suggesting that this formulation does not produce cytotoxic effects under the tested conditions. Keratinocytes represent the predominant cell type in the epidermis; therefore, maintaining their viability is a key indicator of the compatibility of topical formulations with skin tissue [39]. Histological evaluation of ex vivo porcine skin confirmed that skin samples exposed to CP-NE and Blank-NE retained the normal architecture of the epidermis and dermis, showing no signs of tissue alteration or cell damage compared to the negative control treated with physiological saline solution. These findings suggest that the nanoemulsion and its excipients do not induce structural alterations in the skin and, therefore, have a suitable safety profile for dermal application. The good tolerability observed may be related to the well-established dermatological use of the excipients employed in the formulation [40]. Labrasol^®^ and Transcutol^®^ have been widely described as effective solubilizers and permeation enhancers with acceptable safety profiles, while Plurol Oleique^®^ contributes to the stability of colloidal systems and promotes controlled interaction with the skin barrier [25]. Previous studies have reported that topical formulations containing these excipients do not produce significant histological alterations in human or animal skin, findings that are consistent with those observed in the present study [41,42]. These observations are also supported by the evaluation of biomechanical skin parameters following topical application of the CP-NE to the dorsal skin of mice. Measurements of TEWL and SCH did not show statistically significant alterations during the 8 h evaluation period, indicating that the formulation does not impair the barrier function of the stratum corneum. TEWL and SCH are widely used dermatological indicators for assessing potential irritant effects of topical products, as alterations in these parameters may reflect damage or destabilization of the skin barrier [43]. In this study, the transient increase in SCH observed shortly after application likely reflects the moisturizing properties of the formulation. These findings indicate that the nanoemulsion acts as a biocompatible vehicle capable of delivering the drug without compromising skin physiology. However, the HET-CAM assay performed in this study revealed a potential irritant effect upon ocular exposure. Therefore, although CP-NE shows good dermal compatibility, precautions should be taken to avoid contact with the eyes, as irritation may occur under such conditions.

Regarding the therapeutic efficacy, CP-NE produced a marked reduction of the post-surgical inflammation in the mouse model carried out in this study, showing a trend toward improved tissue recovery compared with diclofenac. These observations were supported by histological findings, which revealed faster restoration of normal skin architecture in tissues treated with CP-NE. The semi-quantitative scoring results showed that the CP-NE-treated group presented lower inflammatory and tissue damage scores compared with the untreated control and, under the evaluated conditions, also showed lower histopathological scores than the diclofenac-treated group, suggesting improved tissue recovery and anti-inflammatory activity. In addition, the study with the Blank-NE group confirmed that the vehicle itself did not exert a relevant anti-inflammatory effect. This effect may be attributed to the nanoemulsion delivery system, which can improve local drug availability, promote retention at the site of application, and facilitate interaction between the drug and inflamed tissue compared with conventional topical formulations [44]. These findings are consistent with previous reports demonstrating that nanostructured delivery systems can enhance the anti-inflammatory activity of CP. CP-loaded nanoparticles have been specifically reported to significantly reduce TPA-induced inflammation in murine models [24]. From a macroscopic perspective, animals treated with CP-NE also showed improved wound appearance, characterized by closure of the surgical incision and the presence of only residual scarring at the end of the treatment period. This observation suggests not only effective control of the inflammatory response but also a favorable tissue recovery process.

While the present study demonstrated promising results regarding the development and therapeutic potential of CP-NE, some aspects should be further investigated to strengthen the translational potential of the formulation for veterinary topical therapy. Future studies should include ex vivo permeation experiments using tissues from multiple biological sources in order to better address inter-individual variability in skin permeability. In addition, although the stability studies confirmed adequate physical and chemical stability under the evaluated conditions, longer-term stability studies would be valuable to establish the shelf-life of the formulation under different storage conditions. Furthermore, considering the exploratory nature of the study and in accordance with the principles of the 3Rs (Replacement, Reduction, and Refinement), the in vivo efficacy evaluation was conducted using a relatively limited number of animals. Although significant differences were observed between treatments, studies involving larger sample sizes would further strengthen the statistical robustness and translational relevance of the findings.

## 5. Conclusions

This study demonstrates that nanoemulsion technology is a promising approach for the topical delivery of CP. The developed CP-NE showed suitable physicochemical and biopharmaceutical characteristics for dermal application. Safety assessments confirmed that the formulation is well tolerated, as no cytotoxic effects, histological alterations, or impairment of skin barrier function were observed. Importantly, the in vivo study demonstrated that CP-NE effectively reduced post-surgical inflammation and promoted faster recovery of skin architecture compared with the reference treatment. These findings contribute to the growing body of evidence supporting nanoemulsions as versatile carriers for topical drug delivery and suggest that CP-NE could represent a promising alternative for the localized management of post-surgical inflammation in veterinary medicine. Future studies should focus on evaluating the formulation in additional preclinical settings and exploring its potential applications in other inflammatory conditions requiring localized anti-inflammatory therapy.

## Figures and Tables

**Table 1:** Classification of the groups for anti-inflammatory activity.

	Treatment	Cutting and Suturing	
Group 1	No	No	Negative control
Group 2	No	Yes	Positive control
Group 3	Diclofenac gel	Yes	Commercial treatment Control
Group 4	CP-NE	Yes	Study group
Group 5	Blank-NE	Yes	Blank-NE group

**Table 2:** Final composition of CP-NE.

Components	%
Carprofen (10 mg/g)	
Plurol^®^ oleique CC 497	10
Labrasol^®^	28
Transcutol^®^-P	7
Water	55

**Table 3:** Permeation and prediction parameters of CP-NE through pig skin.

Permeation and Prediction Parameters	Mean ± SD
J*_ss_* (µg/h/cm^2^)	2.052 ± 0.04559
K_p_ (cm/h)	(2.052 ± 0.095) × 10^−4^
Tl (h)	1.096 ± 0.021
P_1_ (cm)	(1.35 ± 0.038) × 10^−3^
P_2_ (1/h)	0.152 ± 0.091
C_ss_ (µg/mL)	0.040 ± 0.002
Q_ret_ (µg/cm^2^)	791.02 ± 15.273

Abbreviations: J_ss_(flux), K_p_ (permeability coefficient), Tl (lag time), P_1_ (vehicle/tissue partition coefficient), P_2_ (diffusion coefficient), C_ss_ (steady-state plasma concentration), Q_ret_ (amount of drug retained in the tissue).

**Table 4:** Semi-quantitative histopathological scoring of skin samples.

Group	Dermal Inflammatory Infiltrate	Epidermal Infiltrate	Dermal Edema	Epidermal Thickening	Dermal Tissue Damage
Positive control	2.33 ± 0.33	1.00 ± 0.01	1.50 ± 0.50	1.70 ± 0.34	2.50 ± 0.16
Negative control	0.50 ± 0.50	0.00 ± 0.00	0.00 ± 0.00	0.00 ± 0.00	0.33 ± 0.00
Diclofenac gel treatment	2.16 ± 0.16	1.66 ± 0.66	1.00 ± 1.00	1.66 ± 0.66	2.16 ± 0.16
CP-NE	1.00 ± 0.00	1.00 ± 0.00	1.00 ± 0.00	0.33 ± 0.00	1.33 ± 0.33
Blank-NE	2.83 ± 0.17	2.42 ± 0.09	1.5 ± 0.50	2.25 ± 0.25	2.83 ± 0.17

## Data Availability

The data presented in this study are available in this article.
